# Built Environments to Support Rehabilitation for People With Stroke From the Hospital to the Home (B-Sure): Protocol for a Mixed Method Participatory Co-Design Study

**DOI:** 10.2196/52489

**Published:** 2023-11-09

**Authors:** Maya Kylén, Jodi Sturge, Ruby Lipson-Smith, Steven M Schmidt, Hélène Pessah-Rasmussen, Tony Svensson, Laila de Vries, Julie Bernhardt, Marie Elf

**Affiliations:** 1 Department of Health Sciences Lund University Lund Sweden; 2 School of Health and Welfare Dalarna University Falun Sweden; 3 Department of Design, Production and Management Faculty of Engineering Technology University of Twente Twente Netherlands; 4 The MARCS Institute for Brain, Behaviour and Development Western Sydney University Westmead Australia; 5 The Florey Institute of Neuroscience and Mental Health Heidelberg Australia; 6 Department of Neurology, Rehabilitation Medicine, Memory Clinic and Geriatrics Skåne University Hospital Malmö Sweden; 7 Department of Clinical Sciences Lund University Lund Sweden; 8 School of Information and Engineering Dalarna University Borlänge Sweden

**Keywords:** stroke rehabilitation, built environment, person-centered, participation, self-efficacy, meaningful activities, accessibility, participatory co-design, good quality and local care

## Abstract

**Background:**

A global trend is to move rehabilitation closer to people's neighborhoods and homes. Still, little attention has been given to how the built environment outside the hospital setting might impact rehabilitation and recovery for stroke survivors.

**Objective:**

The overarching objective of this project is to develop conceptual models of built environments that support stroke rehabilitation and recovery outside the hospital setting. Specifically, the project will explore factors and characteristics of the built environment that support people with stroke and their families and identify innovative built environments that can be designed for local health care. The project will examine facilitators and obstacles for implementing built environmental solutions and evaluate the potential benefits, feasibility, and acceptability.

**Methods:**

The project uses a mixed methods design approach with 3 phases. In phase 1, factors and characteristics of the built environment for rehabilitation will be identified. Based on the results from phase 1, phase 2 will involve co-designing prototypes of environments to support the rehabilitation process for people with stroke. Finally, the prototypes will be evaluated in phase 3. Qualitative and quantitative methods will include a literature review, a concept mapping (CM) study, stakeholder interviews, prototype development, and testing. The project will use multidimensional scaling, hierarchical cluster analysis, descriptive statistics for quantitative data, and content analysis for qualitative data. Location analysis will rely on the location-allocation model for network problems, and the rule-based analysis will be based on geographic information systems data.

**Results:**

As of the submission of this protocol, ethical approval for the CM study and the interview study has been obtained. Data collection is planned to start in September 2023 and the workshops later in the same year. The scoping review is ongoing from January 2023. The CM study is ongoing and will be finalized in the spring of 2024. We expect to finish the data analysis in the second half of 2024. The project is a 3-year project and will continue until December 2025.

**Conclusions:**

We aim to determine how new environments could better support a person’s control over their day, environment, goals, and ultimately control over their recovery and rehabilitation activities. This “taking charge” approach would have the greatest chance of transferring the care closer to the patient's home. By co-designing with multiple stakeholders, we aim to create solutions with the potential for rapid implementation. The project’s outcomes may target other people with frail health after a hospital stay or older persons in Sweden and anywhere else. The impact and social benefits include collaboration between important stakeholders to explore how new environments can support the transition to local health care, co-design, and test of new conceptual models of environments that can promote health and well-being for people post stroke.

**International Registered Report Identifier (IRRID):**

DERR1-10.2196/52489

## Introduction

### Overview

The Built environments to support rehabilitation for people with stroke from the hospital to the home (B-Sure) initiative aims to create conceptual models of environments designed specifically to support the rehabilitation of stroke patients, from hospital settings to their homes. The project addresses the fundamental transformation of health care to provide care and rehabilitation close to the person or at home, that is, good quality and local health care [1-3]. Exploring how the built environment can support these new care policies is important to enhance a person’s potential to live an active life. Surprisingly, little consideration has been given to how the built environment might influence rehabilitation and recovery for people with stroke. Developing new, more supportive environments may be a crucial factor in the success of local health care innovation.

### Background

Stroke is a highly prevalent and debilitating brain injury that causes disability in adults [4,5]. In Sweden alone, an estimated 25,000 people are affected annually [6], while globally, stroke affects over 15 million people yearly, resulting in over 5 million deaths [5]. People with stroke often have long-lasting rehabilitation needs, and recovery is known to be complex, with several care providers involved [7-9]. The effects of stroke can be profound and lead to a range of activity and participation limitations [9-11], such as reduced quality of life, social isolation [12-14], and adverse events such as falls [9,15]. Many patients and families describe the support provided poststroke as poor and not patient-centered [16-18] and report poor engagement in their care and treatment decisions [7,19]. In addition, the role and importance played by the built environment to support rehabilitation outcomes have been largely ignored, especially when patients return home [20,21].

Rehabilitation aims to restore a person’s functional capacity and societal participation after injury [22]. Effective health care services support a person’s independence, participation, and self-directed capacity as people return to the community after a stroke. Recovery after a stroke is best supported when the person and their family or care network feel empowered to take responsibility for their rehabilitation, recovery goals, and activities [22,23].

There is a general need for improvements in the rehabilitation environments. Hospital environments, even specialized rehabilitation hospitals, make patients feel bored and lonely and inhibit independence and control [24-26]. These shared experiences can influence recovery and disempower individuals from leading or engaging in meaningful recovery. Studies show that patient outcomes vary between rehabilitation facilities [27], possibly partially due to the differences in the built environment [28,29]. The design of rehabilitation environments may impact the recovery of people with a stroke, affecting their function in the long term [30]. For society, this can increase disability expenditure and reduce productivity by hampering people's ability to participate fully [26,30]. It is worth noting that in most design strategies in rehabilitation, the built environment has not undergone empirical testing or evaluation.

### New Places for Health Care

Moving care and rehabilitation to peoples’ neighborhoods and in the home is a global development [1]. In Sweden, the reform, “good quality and local health care” is described as a new service [3] with more outpatient care and less but highly specialized inpatient care. This requires an empowered, more self-directed patient with control over their care and rehabilitation. The care must be person-centered and needs to switch from traditional 1-way expert providers to shared decisions with the patient [31,32]. Health care is far from fulfilling the demands of person-centered care and shared decision-making [33,34]. The Swedish government argues that the reform has implications for the building sector as they must be involved in policy discussions to avoid the risk when expensive new inpatient hospital building projects with large climate footprints are realized. At the same time, care and rehabilitation should move closer to patients or into their homes [3].

Developing local health care systems will require innovative approaches to living environments and care provision. These approaches must prioritize providing safe and dignified care, healthy working environments for health care staff, and support for continued independent living despite disability [35]. To achieve these goals, new models of care that promote health and rehabilitation in alternative settings may be necessary, such as rehabilitation hotels, small recovery homes, and day rehabilitation environments. Web-based care and technology, such as telerehabilitation, may also be critical in creating effective transition pathways between different care environments [36-38]. To achieve this, it will be important to develop simple systems for monitoring progress and requesting appointments with health care teams, which can be embedded into the built environment of these care settings.

Our starting point was to ask whether the alternative care environments, between hospital and home, might better support people with rehabilitation needs and avoid the current negative effects of hospital-based rehabilitation. While rehabilitation at home may suit some more mildly affected individuals with stroke and their families, it is impossible for others. Our goal is to explore built environment solutions for individuals who require more support or for individuals unable to readily or immediately go home. This direction aligns with the 2030 Agenda for Sustainable Development to ensure cities are inclusive, safe, resilient, and maintainable and promote well-being for all [39]. We believe the design and development of built environments can support recovery for people with stroke but also benefit others with similar needs and functional challenges, for example, multiple sclerosis or Parkinson and those sensitive to obstacles in their environment, including older people.

Despite the growing trend of rehabilitation at home, earlier research has neglected the role of the environment in this transformation, creating a significant knowledge gap [40]. This gap undermines the effectiveness of rehabilitation at home. There is a pressing need for an in-depth exploration of the environmental factors that impact rehabilitation outcomes, including innovative methods and solutions. An integrative mixed methods approach and participatory design can offer a comprehensive understanding of the importance of the environment for rehabilitation at home. By using these approaches, it will be possible to generate robust evidence on the impact of environmental factors on rehabilitation outcomes, inform the development of guidelines and policies for local care and rehabilitation, and enhance the effectiveness of rehabilitation at home.

### Objectives and Research Questions

B-Sure aims to explore the essential factors in a built environment that supports stroke survivors and their families during rehabilitation. The specific research questions include (1) what are the most important factors in a built environment that supports people with stroke and their families in their rehabilitation process? (2) What do innovative built environments for local health care and rehabilitation look like? (3) What are the significant facilitators and obstacles for implementing various built environmental solutions? (4) How do stakeholders evaluate different built environment solutions regarding their potential benefits, feasibility, and acceptability?

### Theoretical Framework

We will use several theories and frameworks to conceptualize and explore the interaction between a person and the environment. For example, the Person-Environment-Occupation model [41] and the International Classification of Functions [42] show that the environment comprises many facilitating or hindering factors external to the person. These factors include features of the built environment (eg, stairs and doors), natural environment (eg, surfaces outdoors), and objects. The models describe that a good fit between a person’s (P) functional abilities and the demands of environmental factors (E) leads to positive outcomes such as increased independence and overall well-being [43-45]. Hence, to optimize rehabilitation outcomes, it is important to use a person’s environment and be aware of facilitating and hindering factors.

B-Sure is also based on theoretical components of self-efficacy in which the person is seen as capable, with unique experiences, expectations, needs, and resources. Self-efficacy is a key construct from Bandura’s theory of social cognition [46]. It is defined as “people’s beliefs about their capabilities to produce designated levels of performance that influence events that affect their lives.” Self-efficacy beliefs can determine how people feel, think, motivate themselves, and behave concerning their health. For example, self-efficacy influences motivation and health behaviors by determining people’s goals, how much effort they invest in achieving them, and their resilience when faced with difficulties or failure.

In addition, B-Sure is based on the framework of Living-Lab and co-design [47], that is, a close collaboration between stakeholders in the design development process. One of the major challenges in planning and architectural practices today is the communication gap between the design team, the various levels of user groups, and the wide array of specialized consultants in the process.

## Methods

### Study Design

B-Sure has a mixed methods approach [48], including participatory co-design [49,50]. The project has 3 phases. In phase 1 a literature review, concept mapping (CM), and interviews will be done. In phase 2 the results from phase 1 will be used to co-design prototypes of environments to support the rehabilitation process for people with stroke. Finally, phase 3 will evaluate the prototypes obtained with the co-design process. The process will be iterative, enabling knowledge accumulation and increased common understanding between stakeholders and researchers, and contributing to solid knowledge production.

### Phase 1: Identify Factors

#### Overview

Phase 1 involves 3 primary methods of data collection. First, a rapid scoping review [51] will be conducted to synthesize relevant knowledge of essential environmental factors. Second, a participatory mixed methods approach called CM [52] will engage stakeholders in mapping conceptual areas related to the built environment's importance for home rehabilitation. This process will involve collecting qualitative and quantitative data through generated statements, which will then be synthesized and sorted. Finally, interviews with diverse stakeholders will be conducted, covering sociodemographic data and open-ended questions related to identifying crucial factors for promoting the rehabilitation process of people with stroke and their families within the built environment.

#### Participants

Participants from five stakeholder categories will be recruited for the CM and the interviews: (1) people with stroke; (2) relatives or significant others; (3) health care staff; (4) government, including officials responsible for the operation of health care environments and stroke rehabilitation in the region and municipalities; and (5) industry practitioners, including architects and other designers working within the health care contexts and home environments. We will strive to recruit 20 individuals from each category for the CM study and 3-5 people from each category for the in-depth interviews.

People who have had a stroke and their relatives will be recruited locally and regionally through advertising on several portals: hospital notice boards, stroke units, outpatient wards, the STROKE National Association's website, and social media. Participants meet the criteria to participate if they are willing to give written informed consent, have a diagnosed stroke, are 18 years of age and older, or care for someone who has had a stroke. We will use a strategic selection procedure with maximum variation to ensure we get a broad description of experiences from the participants regarding age, gender, time since stroke, ethnicity, and geographical location. Staff with clinical experience in rehabilitation after a stroke will be recruited through the research group's contacts, email lists, and targeted personal emails. We will strive to have a balance between different professionals and regional distribution. We will also recruit representatives from the region, municipality, and real estate owners through our network and collaborators.

#### Analysis

We will use multidimensional scaling analysis and hierarchical cluster analysis for the CM of quantitative data [53] and qualitative content analysis [54] for the interviews and CM. The NVivo software (QSR International) [55] will be used to aid the qualitative analyses and promote the validation and transparency of the coding.

### Phase 2: Co-Design Prototypes or Models of Environments

#### Overview

This aspect of the research will be carried out within the framework of a Living Lab [47] using an iterative and learning co-design approach to engage stakeholders. Building on Phase 1, we will use the well-established Double Diamond method [56] as a guide for the workshop, previously used by the research group [57]. The method includes a three-step process: (1) idea generation, (2) modeling a prototype, and (3) testing and discussing. The research team will facilitate the sessions. Each co-design session is scheduled to continue the work of the previous session. The sessions will be combined with meetings with an advisory committee to guide the progression of the developing prototype. We will use tools and techniques that combine narratives, creativity, and imagination (scenario planning, group discussions, world cafe, individual work, collective sessions, and mock-ups) to ensure we reach all participants based on their characteristics and to guarantee that power is shared within the group.

We will be running 2 co-design processes, 1 in a dense city area and 1 in a rural area. The process will consist of 5 co-design sessions of 3 hours, which will take place over 6 months.

In this process, we will also use a geographic information system (GIS) method [58]. Through the analysis, we can visualize optimal places with the highest possible accessibility for the population. To perform the analysis, we will collect network data for transport systems, information about the population, government thresholds, and rules. Sensitivity analysis of optimal places will be done where the population's place patterns alternate (according to gender, socioeconomic status, and health status). Furthermore, a rule-based modeling method will be used to work out the optimal places where the identified needs from the literature study and the interviews will be used as criteria and matched with environments in the urban area and in the rural area.

#### Participants

Participants from five stakeholder categories (not included in phase 1) will be recruited: (1) persons with stroke; (2) relatives or significant others; (3) health care staff; (4) government, including officials responsible for the operation of health care environments and stroke rehabilitation in the region and municipalities; and (5) industry practitioners, including architects and other designers working within health care contexts and home environments. We will strive to have at least 2-4 people from each group. They will be recruited in the same way as in phase 1.

#### Data Collection

The data will be obtained from (1) notes taken by the team, (2) prototypes produced by each group, and (3) notes taken after the working sessions via a meeting with the research team to share their impressions.

#### Analysis

A qualitative content analysis [54] supported by using NVivo software [55] will be used. To enhance the mixed methods approach of CM a qualitative GIS method [58,59] will be used to spatially analyze and contextualize the CM results. Open access Swedish spatial data (ie, street network files and geocoded health services) will be triangulated with the CM results using qualitative GIS methods to provide a layered, grounded theory understanding of the meaning and experiences of the stakeholders. A qualitative GIS approach offers a deeper understanding of the context and meaning of the situations that influence person-place interactions and the social factors that relate to the perception and interactions.

### Phase 3: Evaluation of the Prototypes

#### Overview

The third phase will evaluate the different prototypes or models of different environmental solutions according to benefits, feasibility, and acceptability. This is a matter of observing future user and stakeholder discussions when confronted with the prototypes, aiming to identify possibilities and challenges with the prototypes.

A combination of think-aloud and focus group interviews will be used. The think-aloud method is frequently used to reveal views from users when they encounter potential solutions [60]. In general, this method aims to capture a systematic process of thinking aloud and analyze this process to gain a deeper understanding of the feasibility of the developed models or prototypes. The sessions will be filmed to allow for transcription of the communications and permit us to associate them with the prototypes.

#### Participants

A sample of potential users and stakeholders will be recruited. We will base the sample on the 5 stakeholder groups and recruit 2-3 people from each group.

#### Analysis

The transcriptions will be coded to identify possibilities and challenges with the prototypes and content analysis will be used [54].

### Patient and Public Involvement

B-Sure study aims to achieve active engagement and empowerment of persons with stroke, health care professionals, decision makers, and other stakeholders by developing partnerships that emphasize equal power while acknowledging different roles and responsibilities. The collaborative nature of the research design supports a strong patient and public involvement approach. We are actively working in collaboration with stroke organizations and stroke units where the research questions for the study have emerged. This ensures that the research aligns with the needs and perspectives of stroke survivors, their families, and the wider community. During the initial phase, individuals with stroke, family members, and relevant stakeholders, including representatives from stroke organizations and stroke units, will contribute their insights and lived experiences to identify key factors and characteristics of the built environment for rehabilitation. The co-design phase will adopt a participatory approach, actively involving stroke survivors, their families, stakeholders, and representatives from stroke organizations and units as partners in developing prototypes for rehabilitation-supportive environments.

To generate and disseminate knowledge, an iterative Integrated Knowledge Translation approach will be used, with participants and researchers functioning as cocreators. Practical implications of cocreators include the terms “knowledge user” and “stakeholder,” as defined by the International Association for Public Participation Spectrum of Public Participation [60]. The study will evaluate the level of participation using this framework [61].

### Dissemination

Our participatory approach involves engaging knowledge users and researchers as partners throughout the study. We will create knowledge products that feature the review results, interviews, and co-design process. These products will include recommendations for improvements in stroke care and presentations for health care leaders, clinical teams, and policy makers. Additionally, we will produce traditional academic outputs like conference presentations and publications.

### Ethical Considerations

The study has ethics approval from the Swedish Ethical Review Authority (2023-02337-01 and 2022-04231-01). The project will adhere to ethical guidelines outlined in the Helsinki Declaration. Informed consent will be obtained. Written and oral information on the study purpose and what participation would entail will be given before the informed consent. They will also be informed about confidentiality and their right to withdraw from the study at any time. The design of B-Sure is inherent, iterative, and dynamic. This means participants will be able to keep discussions about ethical issues along the way.

## Results

The B-Sure is a 3-year project which started in January 2022 and is planned to continue until December 2024. The project was funded in November 2022, ethical approval was obtained in March 2023. Data collection is planned to start in September 2023, and the workshops later in the same year. The scoping review is ongoing from January 2023. The CM study is ongoing and will be finalized in the spring of 2024. We expect to finish the data analysis in the second half of 2024.

## Discussion

### Principal Results and Social Benefit

We aim to determine how new environments could better support a person’s control over their day, environment, goals, and ultimately control over their recovery and rehabilitation activities. This “taking charge” [22] approach would have the greatest chance of transferring the care closer to the patient's home. By co-designing with multiple stakeholders, we aim to create solutions with the potential for rapid implementation. The project’s outcomes may be scaled up and target other persons with frail health after a hospital stay or older persons in Sweden and elsewhere.

B-Sure will provide knowledge about how buildings and environments in society can support the new care landscape with more care and rehabilitation outside the hospital. The project can thus contribute to improved, strategic, and maintainable planning of environments in society. We will examine this issue from the perspective of the needs of people with stroke in their recovery and rehabilitation phases. Stroke is common, and the affected individuals have complex health needs. As acute care shortens, rehabilitation and recovery will increasingly happen in the community or at home.

We will use Living-Lab and participatory co-design, where the users (patients, relatives, and professionals) and other key stakeholders such as decision makers in the health service and municipalities, architects, and building planners will be involved. There is a need for collaboration between the users, the building sector, health care, regions, and municipalities to develop innovative and maintainable environments that can support the transition to local health care. Overall, there is a great need for investments in health care environments, which affects the resource space for care. Poor design that is not supportive of the end users, entails significant costs for the patients, the government, and health care and risks contributing to large climate footprints as the buildings reduce the chance of being maintainable. In addition, we know that traditional planned building projects are often stuck in conventional thinking and risk cementing old care structures and obstacles between the care providers while innovative solutions are needed.

It is still uncommon for more developed, innovative collaboration between the stakeholders when planning and developing facilities [62]. The Swedish government has drawn attention to this [63]. It intends to map and analyze how these investment projects related to the ongoing development of health care at a national level and how facilities can contribute to the national development of health care and increased socioeconomic efficiency.

We expect that the knowledge produced by B-Sure is critical to fill this gap. The impact and social benefits include (1) Collaboration between important stakeholders (patients, health care, and building sector) to explore how new environments can support the transition to local health care. (2) Co-design and test new conceptual models of environments that can promote health and well-being of people poststroke. (3) Conduct a participatory design process that can create conceptual models where the built environment is included as an important part of rehabilitation. (4) Stimulate the establishment of a strong national network that can strengthen the development of high-quality rehabilitation environments.

With the B-Sure approach, new knowledge and results will be disseminated locally, nationally, and internationally by our broad stroke rehabilitation and architecture network. We will initiate and participate in knowledge transition activities such as conferences and seminars. Most importantly, we have designed the project to include persons with stroke, relatives, staff, and actors from society, enabling rapid user feedback and the spread of results to a wider public. We will initiate and be involved in knowledge transition activities such as conferences and seminars as contributing to a collaboration between health care, region, and municipalities is the goal to generate knowledge and bilateral development.

### Strengths and Limitations of This Study

The study aims to examine the specific requirements of individuals with subacute stroke during their recovery and rehabilitation phases, emphasizing the crucial role of built environments in facilitating innovative care approaches beyond the hospital setting.

This project will foster collaboration among key stakeholders, including stroke survivors, health care professionals, the building sector, and policymakers. This collaborative effort will explore how the built environment can effectively support the transition toward the new health care landscape with local health care, thereby driving the development of optimal rehabilitation environments.

The research findings will not only enhance the knowledge base for individuals with stroke, health care providers, building sectors, policymakers, and educational institutions but also underscore the critical significance of the built environment.

These findings will pave the way for introducing groundbreaking solutions in health care services and communities. Although implementing these solutions may encounter challenges, the research team ensures maximum buy-in by actively involving key representatives from relevant institutions and units as integral research team members. This approach ensures a comprehensive and well-rounded perspective in addressing the complexities of optimizing the built environment for stroke rehabilitation.

## Appendix

Multimedia Appendix 1 Peer-review report by Formas, Sweden.

## Abbreviations

B-Sure: Built environments to support rehabilitation for people with stroke from the hospital to the home
CM: concept mapping
GIS: geographic information system

## Data Availability

The data sets generated and analyzed during this study are available from the corresponding author on reasonable request.
