# Supplementary material for: Built Environments to Support Rehabilitation for People With Stroke From the Hospital to the Home (B-Sure): Protocol for a Mixed Method Participatory Co-Design Study
Source: JMIR Res Protoc. 2023 Nov 9;12:e52489. doi: 10.2196/52489 (PMC10667985; doi:10.2196/52489)
Supplement: Multimedia Appendix 1 [file resprot_v12i1e52489_app1.pdf]

FORMAS

Beredningsgruppens yttrande

**2021-01455**	**Marie Elf**	**Beredningsgrupp: Brg2108**

**Utlysningsnamn:** Årliga öppna utlysningen 2021	**Bidragsform:** Forskningsprojekt
**Projekttitel (svenska):** Byggda miljöer för att stödja rehabilitering för patienter med stroke - Från sjukhuset till hemmet (B Sure)	**Sökt inriktning:** Forskarinitierad

Vetenskaplig frågeställning 6

1 - Insufficient, 2 - Poor, 3 - Acceptable, 4 - Good, 5 - Very Good, 6 - Excellent, 7 - Outstanding

Metod och genomförande 5

1 - Insufficient, 2 - Poor, 3 - Acceptable, 4 - Good, 5 - Very Good, 6 - Excellent, 7 - Outstanding

Vetenskaplig kompetens 6

1 - Insufficient, 2 - Poor, 3 - Acceptable, 4 - Good, 5 - Very Good, 6 - Excellent, 7 - Outstanding

Frågeställningens samhällsnytta och kommunikation av resultat 6

1 - Insufficient, 2 - Poor, 3 - Acceptable, 4 - Good, 5 - Very Good, 6 - Excellent, 7 - Outstanding

Slutbedömning (max 1 000 tecken inklusive mellanslag) 6

1 - Insufficient application, 2 - Poor application, 3 - Acceptable application, 4 - Good application, 5 - Very good application, 6 - Excellent application, 7 - Outstanding application

This is a novel, scientifically and societally important and novel project analyzing the potential and requirements of built environments to support the rehabilitation of patients suffering from a stroke. The research plan is excellently thought out and laid out, with many details about the overall research design, data to be collected in partnership with different stakeholders and using Living Labs, as well as appropriate data analysis. However, the proposed research requires ethical considerations. Overall, is particularly relevant to include a mixed-method design, including participatory co-design and prototype-testing and strong stakeholder participation. The societal contributions of the project are extensive, both from the point of view of creating healthier and more livable urban environments and also of improving care for socially and healthy vulnerable residents. Last, the overall group has extensive fundraising, supervision, and publication experience, especially so in the context of stroke recovery.
